# Comparing Outcomes of ACL Reconstruction with and Without Cyclic Graft Loading: A Two-Year Minimum Follow-Up Study

**DOI:** 10.3390/jcm15093318

**Published:** 2026-04-27

**Authors:** Jérôme Murgier, Thibaut Tourcher, Sonja Cabarkapa

**Affiliations:** 1 Clinique Aguiléra—Ramsay Santé, 21 Rue de l’Estagnas, 64200 Biarritz, France; 2 Orthopedic Department, Clinique Universitaire du Sport, CHU Toulouse, 31000 Toulouse, France; 3 St Vincent’s Hospital, Melbourne, VIC 3065, Australia

**Keywords:** anterior cruciate ligament, graft tensioning, knee rehabilitation, knee laxity

## Abstract

**Background:** The necessity of cyclic graft loading during anterior cruciate ligament (ACL) reconstruction remains debated. This study aimed to compare clinical outcomes between patients undergoing ACL reconstruction with and without cyclic graft loading. **Methods:** A retrospective comparative study was conducted involving 216 patients who underwent ACL reconstruction between 2021 and 2022. Patients were divided into two groups: those whose grafts were cyclically loaded intraoperatively (*n* = 104) and those who did not undergo cyclic loading (*n* = 112). All surgeries used hamstring autografts and similar fixation techniques. Outcomes, including graft re-rupture rates, knee laxity (measured by arthrometer, Rolimeter^®^), and patient-reported outcomes, were evaluated at a minimum follow-up of two years. **Results:** The demographics of both groups were comparable. Re-rupture rates were 2.5% in the cyclic loading group and 2.9% in the non-cyclic loading group (*p* = 0.78). Mean side-to-side laxity difference in anterior tibial translation measured by Rolimeter^®^ was 1.1 mm ± 0.6 in the cyclic loading group and 1.2 mm ± 0.7 in the non-cyclic loading group (*p* = 0.39). No significant differences in Lysholm or IKDC scores were observed between groups. **Conclusion:** In this retrospective, non-randomized cohort, no statistically significant differences were detected between ACL reconstruction performed with or without cyclic graft loading. These findings should be interpreted with caution, given the potential for temporal confounding. Further prospective, randomized studies are required to confirm these results.

## 1. Introduction

Level of Evidence: III, retrospective comparative study.

The anterior cruciate ligament (ACL) is one of the primary stabilizing structures of the knee joint, preventing excessive anterior translation of the tibia relative to the femur and contributing to rotational stability [1]. In addition to its mechanical role, the ACL also plays an important function in proprioception, providing sensory feedback that contributes to neuromuscular control of the knee [2]. The loss of this function following injury may further impair joint stability and coordination [3], even in the absence of gross mechanical laxity, as well as an increased risk of secondary injuries such as meniscal tears and cartilage damage [4]. ACL injuries represent a significant burden in orthopaedic practice worldwide, with increasing incidence, particularly among young and active individuals [5]. The rise in participation in pivoting sports (e.g., basketball), combined with higher physical demands and earlier return-to-sport expectations, has contributed to a growing number of ACL reconstructions performed annually [6]. The management of ACL injuries has evolved considerably over the past decades, with continuous improvements in surgical techniques, graft selection, fixation devices, and rehabilitation protocols [7]. A recent study of ACL reconstruction using a bone–patellar tendon–bone (BPTB) allograft compared with a hamstring tendon autograft showed similar results at evaluation, but the allogenic BPTB enabled an earlier return to sporting and daily life activities [8]. Contemporary research also highlights that femoral fixation employing cortical buttons conferred less knee laxity than interference screws for graft fixation in ACL reconstruction [9].

Despite these advances and the growing knowledge base, restoring normal knee biomechanics and preventing long-term complications remains challenging, and a proportion of patients continue to experience residual symptoms or suboptimal outcomes [10]. As a result, considerable research efforts have focused on refining surgical techniques and identifying modifiable intraoperative factors that may influence graft behavior and clinical outcomes [11]. While recent in vivo animal studies aim to finesse the biomechanical rationale into clinically relevant practice [12], ACL reconstruction remains the gold standard treatment for restoring knee stability and functionality in patients with ACL tears [12,13,14]. Certain aspects of the surgical procedure remain contentious [15,16]. One such debate surrounds the practice of cyclic graft loading during the intraoperative phase of ACL reconstruction [12]. The concept of graft preconditioning through cyclic loading is based on the viscoelastic properties of tendon grafts. Cyclic loading refers to the repeated flexion and extension of the knee before final graft fixation, with the goal of preconditioning the graft. Tendon tissues demonstrate creep and stress relaxation when subjected to sustained mechanical loading, potentially leading to graft elongation during the early postoperative period [17]. For this reason, many surgeons perform repeated cycles of knee flexion and extension intraoperatively before final graft fixation in order to remove slack and stabilize graft length [18]. Experimental biomechanical studies have suggested that cyclic loading may eliminate slack in the graft and ensure tautness, minimize viscoelastic creep, a phenomenon where the graft lengthens under sustained load and reduce the risk of postoperative graft elongation and subsequent instability [19]. However, these findings have not always translated into consistent clinical benefits. In recent years, advances in fixation devices, particularly adjustable-loop suspensory fixation systems, have improved the ability to maintain graft tension throughout the range of knee motion [20]. As a consequence, the necessity of performing systematic cyclic loading during ACL reconstruction has been questioned.

Some studies suggest that most graft elongation occurs during the first few cycles of motion, with minimal additional changes thereafter, raising doubts regarding the benefit of prolonged cyclic loading protocols [19]. Despite these considerations, clinical evidence evaluating the impact of cyclic loading on patient outcomes remains limited and heterogeneous, with most studies focusing on biomechanical outcomes rather than clinically relevant endpoints and a lack of high-qualitative comparative clinical studies.

This study aimed to evaluate the clinical necessity of cyclic graft loading by directly comparing outcomes in patients who underwent ACL reconstruction with and without this practice. By analysing key metrics such as re-rupture rates, knee laxity, and functional scores, this investigation seeks to provide evidence-based recommendations for optimizing surgical protocols in ACL reconstruction.

The hypothesis was that outcomes would be similar between the two groups. More specifically, it was hypothesized that the omission of cyclic graft loading would not result in increased postoperative knee laxity, higher graft failure rates, or inferior patient-reported functional outcomes at a minimum two-year follow-up. Given the evolution of fixation devices and improved intraoperative tensioning techniques, it was assumed that modern surgical constructs would maintain graft stability independently of intraoperative preconditioning manoeuvres.

In addition, it was anticipated that eliminating cyclic loading could offer practical advantages without compromising clinical outcomes. These potential benefits include reduced operative time, simplified surgical workflow, and improved reproducibility of the technique across different surgical settings. Therefore, the study aimed not only to assess clinical equivalence between the two approaches but also to explore whether cyclic loading could be safely omitted as part of a streamlined and evidence-based surgical protocol.

## 2. Materials and Methods

### 2.1. Study Design and Population

A retrospective, non-randomized comparative study was carried out including all patients who underwent ACL reconstruction between January 2021 and December 2022 at a single institution. Patients were identified through a prospectively maintained surgical database and group allocation was based on a temporal change in surgical practice; thus, this is a non-randomized study with some potential residual confounding. Eligibility criteria included isolated ACL rupture treated with primary reconstruction using a hamstring autograft and availability for a minimum follow-up of 24 months. Exclusion criteria were multi-ligamentous knee injuries, prior surgery on the ipsilateral or contralateral knee, revision ACL reconstruction, and incomplete clinical data. Of the 340 eligible patients, 216 consented to participate and were included in the final analysis. The mean follow-up was 28 months (±8). Preconditioned stretching was performed during graft preparation for each patient.

**Figure 1:**
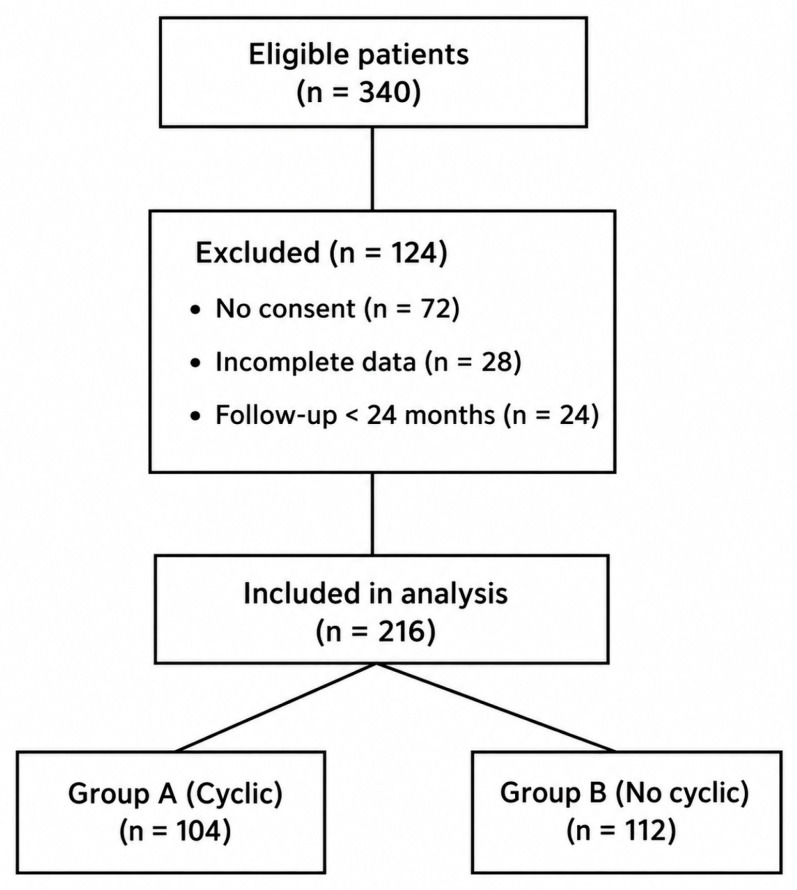
Patient flow diagram.

Patients were divided into two groups according to intraoperative technique (Figure 1):

- Group A (Cyclic Loading): grafts were preconditioned with 20 cycles of knee flexion-extension before final fixation (*n* = 104).
- Group B (No Cyclic Loading): grafts were fixed immediately after tensioning without cyclic loading (*n* = 112).

This grouping reflects a change in surgical practice that occurred in January 2022. Selection bias was considered by ensuring the groups were comparable and the sample was representative. Confounding was also minimized by ensuring that the same experienced senior surgeon performed all surgeries for participants in both groups; thus, there was no difference in surgical expertise. All patients were instructed to follow the same rehabilitation protocol, and despite a temporal difference in the technique, the other aspects of perioperative care did not change over time. No a priori power analysis was performed due to the retrospective nature of the study. Additionally, this is an exploratory study; thus, a formal power calculation would have relied on speculation. Hence, the study was designed to generate estimates for future confirmatory work. Finally, a linear regression model for laxity confidence intervals indicated true equivalence as part of a post hoc analysis.

### 2.2. Outcome Measures

The primary endpoint of this study was the comparison of knee side-to-side laxity measurements between the two groups. This was done using a manual arthrometer (Rolimeter^®^, Aircast Europe, Neubeuern, Germany) to assess anterior tibial translation (mm). This arthrometer has been shown to be reliable and reproducible [1,21,22]. Measurements were repeated three times for each knee, with the knee positioned at 25° of flexion while applying a maximum manual traction. The mean of the three values was used in the analysis. A dry run was performed first for each patient, as much as possible, to ensure that the patient was relaxed. The secondary endpoint was the comparison of the re-rupture rate between the two groups. It was defined as a complete tear of the reconstructed ACL, confirmed by clinical examination and imaging [23]. Finally, the last endpoint was the comparison of Patient-Reported Outcome Measures between the two groups using Lysholm and IKDC scores [24,25].

All these measurements were performed in clinics at a minimum of 2 years after surgery. Patients were asked to fill in the questionnaires, and a surgeon evaluated knee laxity with a Rolimeter^®^.

### 2.3. Surgical Technique

All surgeries were performed arthroscopically by the same experienced orthopaedic surgeon. Hamstring tendon autografts were harvested and prepared into quadrupled constructs [26]. The semitendinosus tendon was harvested through a standard anteromedial incision and prepared as a quadrupled graft construct. Graft diameter was measured using a sizing block prior to implantation. Femoral tunnel drilling was performed using an anteromedial portal technique to achieve an anatomic femoral footprint. Suspensory adjustable-loop fixation devices were used on both the femoral and tibial sides. Final graft fixation was performed with the knee positioned at approximately 20° of flexion while applying manual tension to the graft [27].

In Group A, cyclic loading was performed by flexing and extending the knee from 0° to 90° for 20 cycles before final fixation. In Group B, the grafts were fixed immediately after tensioning without cyclic loading. These two groups were not randomized; instead, they reflect a change in practice when the surgeon stopped cyclic loading in January 2022.

### 2.4. Statistical Analysis

Statistical analysis was performed by the authors using SPSS software (version 27.0). Continuous variables were expressed as mean ± standard deviation and assessed for normality using the Shapiro–Wilk test. Normally distributed variables were compared using independent t-tests, while non-normally distributed variables were analysed using non-parametric tests when appropriate. Categorical variables were analysed using chi-square or Fisher’s exact tests depending on expected frequencies. To improve robustness, exploratory subgroup analyses were performed to consider potential confounding factors such as age, sex, and time from injury to surgery. Although the study was not powered for these exploratory subgroup analyses, they were included to ensure consistency of the main findings across different patient profiles.

A *p*-value of less than 0.05 was considered statistically significant. Confidence intervals (95%) were calculated for primary outcomes to better estimate the precision of the observed differences. Missing data were handled using complete-case analysis, as the proportion of missing values was minimal and unlikely to influence the results.

### 2.5. Ethical Considerations

This study was conducted in accordance with the principles of the Declaration of Helsinki. All patients provided informed consent for the use of their anonymized clinical data for research purposes. Data confidentiality and patient privacy were strictly maintained throughout the study.

### 2.6. Data Collection and Follow-Up

Clinical and demographic data were extracted from electronic medical records and cross-checked with operative reports to ensure accuracy. Follow-up assessments were conducted during routine postoperative visits at a minimum of two years after surgery. In addition to in-person evaluations, some patient-reported outcome measures were collected via standardized questionnaires completed during clinic visits.

Particular attention was given to ensuring consistency in measurement conditions. The same equipment (Rolimeter^®^) and standardized testing position were used for all patients. The surgeon conducting the assessments had prior experience in knee laxity evaluation and followed a predefined protocol to minimize inter-observer variability.

## 3. Results

### 3.1. Study Population and Follow-Up

A total of 216 patients were included in the final analysis, with 104 patients in the cyclic loading group (Group A) and 112 in the non-cyclic loading group (Group B). The overall follow-up duration was 28 ± 8 months, with no significant difference between groups. Baseline demographic characteristics were comparable between groups, with no statistically significant differences in age, sex distribution, body mass index, or time from injury to surgery (Table 1). These similarities support the internal validity of the comparative analysis.

### 3.2. Knee Laxity

The primary outcome measure, side-to-side anterior tibial translation assessed with a Rolimeter^®^, demonstrated comparable results between the two groups. The mean side-to-side laxity was 1.1 ± 0.6 mm in Group A and 1.2 ± 0.7 mm in Group B (*p* = 0.39). The distribution of laxity values was similar in both groups, with the majority of patients demonstrating less than 3 mm of side-to-side difference, indicating satisfactory mechanical stability.

When stratified by age and sex, no clinically relevant differences were observed, and the absence of an effect of cyclic loading remained consistent across subgroups. Additionally, there was no observable correlation between time from injury to surgery and postoperative laxity values (Table 2).

### 3.3. Re-Rupture Rates

The overall graft re-rupture rate was low at 2.7% across the entire cohort. In Group A, 2 patients (2.5%) experienced graft failure, compared to 3 patients (2.7%) in Group B. This difference was not statistically significant (*p* = 0.78). All re-ruptures occurred within the first 18 months following surgery and were associated with return to pivoting sports activities. No clustering of failures related to surgical technique or patient characteristics was identified.

### 3.4. Functional Outcomes

Patient-reported outcomes were high and comparable between groups. The mean Lysholm score was 92.1 ± 4.3 in the cyclic loading group and 91.8 ± 4.6 in the non-cyclic loading group (*p* = 0.67). Similarly, the IKDC subjective score averaged 88.5 ± 5.1 in Group A and 88.3 ± 5.4 in Group B (*p* = 0.74). The distribution of scores indicated that the majority of patients achieved good to excellent outcomes, with no significant ceiling or floor effects observed.

No significant associations were found between functional scores and demographic variables. Both groups demonstrated comparable levels of return to daily activities and sports participation, although this was not formally quantified.

## 4. Discussion

The findings of this retrospective, non-randomized study contribute to the ongoing debate surrounding the necessity of cyclic graft loading during ACL reconstruction. By demonstrating no significant differences in laxity measurements and clinical outcomes between patients undergoing cyclic loading and those who did not, this research challenges the traditional notion that cyclic loading is indispensable for optimal surgical results and to avoid graft secondary laxity. However, given the methodological limitations of this study, this proposed hypothesis requires further consideration.

Cyclic loading is theorized to precondition the graft, eliminating slack and mitigating viscoelastic creep, thereby enhancing graft stability [28]. However, biomechanical studies have indicated that the majority of graft elongation occurs during the initial cycles of knee flexion and extension, with minimal changes observed thereafter [28]. A notable study by Jiang et al. [19] examined the elongation of four-strand hamstring tendon autografts during anterior cruciate ligament (ACL) reconstruction. The researchers found that the graft lengthened significantly during the first 30 cycles of knee flexion and extension, after which the elongation plateaued. Specifically, the median elongation from 30 to 40 cycles was only 0 mm, indicating that most elongation occurred early in the cycling process. This suggests that the majority of graft elongation happens during the initial cycles, with minimal changes thereafter. Another study by Tanabe et al. [29] compared graft length changes among five different anatomic single-bundle ACL reconstruction approaches. The results showed significant differences in graft length changes during knee flexion-extension among the different grafts, with some grafts reaching a plateau in length change after a certain number of cycles. This further supports the notion that graft elongation is most pronounced during the initial cycles of knee motion. These studies collectively indicate that graft elongation predominantly occurs during the initial cycles of knee flexion and extension, with minimal changes observed thereafter. This raises additional questions about the benefits provided by cyclic loading beyond these initial cycles. On the other hand, studies show that cyclic knee motion leads to significant graft elongation, indicating that cyclic loading could potentially compromise graft vitality. Moreover, a study by Spierings et al. [30] highlighted that altered graft mechanical properties, such as decreased stiffness, could lead to knee instability and the early onset of osteoarthritis. This underscores the importance of appropriate graft tensioning over cyclic loading in achieving optimal clinical outcomes. Furthermore, Brophy et al. [31] investigated the effects of short-duration low-magnitude cyclic loading versus immobilization on tendon-bone healing after ACL reconstruction in a rat model. They concluded that such cyclic loading was not detrimental to the strength of the healing tendon-bone interface but was associated with greater inflammation and less bone formation in the tunnel. These findings suggest that the benefits of cyclic loading may be limited and context-dependent. To our knowledge, there is a paucity of studies demonstrating that cyclic loading is beneficial for improving ACL reconstruction outcomes. Furthermore, there is no consensus on the ideal tension that should be applied to the graft [32,33].

### 4.1. Clinical Relevance

Clinically, the relevance of cyclic loading remains questionable. Our results suggest that the routine use of cyclic loading protocols during surgery may not provide a clinical advantage [2], but their interpretation needs to remain measured in terms of the retrospective and non-randomized study design. The underlying reasons why cyclic loading may fail to improve outcomes could be multifactorial. One explanation is that initial graft tension and proper fixation techniques [9,27] likely play a more crucial role in long-term stability than repeated cycles of knee motion during surgery. Also, ACL surgery outcomes are influenced by multiple factors. Biological factors such as graft incorporation, remodelling, addition of lateral tenodesis, and patient rehabilitation protocols might exert a stronger influence on graft integrity and knee function than mechanical preconditioning alone. Consequently, while cyclic loading might simulate early knee movement and help identify mechanical slack in the graft intraoperatively, it appears insufficient to reduce knee laxity or prevent graft failure on its own [34,35]. Future efforts should focus on optimizing graft fixation methods, biological augmentation, and postoperative rehabilitation to improve long-term outcomes following ACL reconstruction.

### 4.2. Limitations

Despite its strengths, this study has several limitations. The retrospective design inherently introduces potential biases, as patient allocation to groups was not randomized. Additionally, the follow-up period of two years, while sufficient for assessing short- to mid-term outcomes, may not capture long-term differences in graft durability or knee function. The fact that surgical practice changed in January 2022, with surgeons no longer tensioning grafts, also poses a limitation. Future studies should aim to include randomized controlled trials with longer follow-up periods to validate these findings and provide more robust evidence. Furthermore, this study did not assess certain biomechanical parameters, such as graft tension during fixation or changes in graft properties over time. These parameters could provide valuable insights into the underlying mechanisms that drive the observed outcomes.

## 5. Conclusions

In this retrospective, non-randomized cohort, no statistically significant differences were detected in knee laxity, graft survival, or patient-reported outcomes at a minimum two-year follow-up. These findings question the need for cyclic loading but should be interpreted with caution, given the potential for temporal confounding. Further prospective, randomized studies are required to confirm these results.

## Figures and Tables

**Table 1:** Patient Demographics.

Variable	Group A (Cyclic Loading) (*n* = 104)	Group B (No Cyclic Loading) (*n* = 112)	*p*-Value
Age (years)	28.4 ± 6.7 [27.112–29.688]	29.1 ± 6.9 [27.822–30.378]	0.48
Male (%)	62	65	0.71
Female (%)	38	35	0.71
BMI (kg/m^2^)	23.5 ± 3.1 [22.904–24.096]	23.7 ± 3.4 [23.070–24.330]	0.63
Time from injury (weeks)	14.8 ± 5.2 [13.801–15.799]	15.1 ± 5.4 [14.100–16.100]	0.79

**Table 2:** Clinical Outcomes.

Outcome	Group A (Cyclic Loading)	Group B (No Cyclic Loading)	*p*-Value
Re-rupture rate (%)	2.5	2.9	0.78
Rolimeter^®^ (mm)	1.1 ± 0.6 [0.985–1.215]	1.2 ± 0.7 [1.070–1.330]	0.39
Lysholm Score	92.1 ± 4.3 [91.274–92.926]	91.8 ± 4.6 [90.948–92.652]	0.67
IKDC Score	88.5 ± 5.1 [87.520–89.480]	88.3 ± 5.4 [87.300–89.300]	0.74
Operative time (min)	72.6 ± 9.1 [70.851–74.349]	65.4 ± 8.3 [63.863–66.937]	<0.001

## Data Availability

The data presented in this study are stored in a password-secured repository and are not publicly available due to patient privacy and confidentiality requirements.
